# Prognostic value of lymph node ratio after neoadjuvant chemotherapy for gastric cancer: a systematic review and meta-analysis

**DOI:** 10.3389/fonc.2026.1755605

**Published:** 2026-03-02

**Authors:** Siyan Liu, Xiang Da, Lihong Shu, Qian Huang, Maolin Zhang, Ruyan Wei, Lijia Yang, Kun Du

**Affiliations:** 1 Department of Clinical Laboratory, First Affiliated Hospital of Yangtze University, The First People's Hospital of Jingzhou, Jingzhou, Hubei, China; 2 College of Health, Medicine and Wellbeing, The University of Newcastle, Newcastle, NSW, Australia; 3 Seventh People’s Hospital of Chengdu, Chengdu, Sichuan, China; 4 Chengdu Medical College, Chengdu, Sichuan, China; 5 Dazhou Vocational and Technical College, Dazhou, Sichuan, China

**Keywords:** gastric cancer, lymph node ratio, meta-analysis, neoadjuvant chemotherapy, overall survival (OS)

## Abstract

**Background:**

The prognostic value of the lymph node ratio (LNR) in gastric cancer treated with neoadjuvant chemotherapy (NACT) remains unclear, as conventional pathological nodal assessment after neoadjuvant therapy may be affected by treatment-induced changes. This meta-analysis evaluated whether LNR retains prognostic significance in patients undergoing neoadjuvant chemotherapy followed by curative gastrectomy.

**Methods:**

PubMed, MEDLINE, CENTRAL, Web of Science, Embase, and ClinicalTrials.gov were searched from inception to October 20, 2025. Retrospective cohort studies reporting hazard ratios (HRs) for survival outcomes based on LNR after NACT were included. Overall survival was the primary outcome; disease-free survival was a secondary outcome. Prespecified subgroup analyses were performed by LNR cutoff (≥0.30 *vs <*0.30) and neoadjuvant regimen (chemotherapy *vs* chemo–immunotherapy).

**Results:**

Seven studies involving 2,437 patients met inclusion criteria. Six studies reported overall survival, one study reported disease-specific survival. High LNR was strongly associated with poorer overall survival (OS)(HR = 3.48, 95% CI: 2.27–5.33) and inferior disease-free survival(DFS) (HR = 1.94, 95% CI: 1.24–3.06). Subgroup analysis showed a significant interaction by LNR cutoff (p = 0.03), but no interaction by neoadjuvant treatment modality (p = 0.49). Sensitivity analyses confirmed the stability of results.

**Conclusions:**

Higher LNR is a strong and independent predictor of poor survival after neoadjuvant chemotherapy in gastric cancer. LNR may improve postoperative risk stratification beyond pathological TNM staging after neoadjuvant therapy, but prospective studies are required to establish optimal cut-offs and determine how LNR should be integrated into clinical decision-making.

**Systematic review registration:**

https://www.crd.york.ac.uk/prospero/, identifier CRD420251233582.

## Introduction

Gastric cancer is the fifth most common malignancy and the fourth leading cause of cancer-related mortality worldwide, with an estimated 1.09 million new cases and 768,000 deaths in 2022 (1). For patients with locally advanced disease, neoadjuvant chemotherapy (NACT) has become an essential component of standard care, supported by pivotal trials such as MAGIC (2) and FLOT4 (3) that demonstrated significant survival benefits compared with surgery alone (4). More recently, the therapeutic landscape has further evolved with the exploration of immune checkpoint inhibitors in the perioperative setting, as seen in major trials like KEYNOTE-585 (5) and MATTERHORN (6), highlighting the increasing complexity of post-treatment prognostic assessment (7). Despite these therapeutic advances, long-term outcomes remain suboptimal, particularly among patients presenting with substantial lymph node involvement at diagnosis (8). This underscores the continued need for accurate postoperative prognostic assessment to guide individualized treatment strategies and identify those at high risk of recurrence (9).

Conventional pathological nodal status after neoadjuvant therapy is determined solely by the absolute number of metastatic lymph nodes. This approach presents notable challenges in the post-NACT context (10). Cytotoxic treatment can eradicate micrometastatic disease, induce stromal fibrosis, and reduce nodal tumor burden, occasionally converting node-positive disease to node-negative status (11). Furthermore, variability in the extent of lymphadenectomy and the total number of lymph nodes retrieved may compromise the accuracy of ypN classification and contribute to marked stage migration (12).

The lymph node ratio—defined as the proportion of metastatic to examined lymph nodes—has emerged as a promising alternative metric that may overcome these limitations (13). By integrating both nodal tumor burden and the adequacy of pathological evaluation, The lymph node ratio(LNR) provides more consistent prognostic stratification across heterogeneous surgical and pathological practices (12). Although previous meta-analyses have demonstrated that the LNR serves as an independent prognostic indicator for postoperative survival across various cancers including colorectal cancer (14), non-small cell lung cancer (15), its prognostic significance in patients with gastric cancer undergoing neoadjuvant chemotherapy remains uncertain (16).

Given the widespread adoption of NACT and the clinical need for robust post-treatment risk stratification tools, clarifying the prognostic relevance of LNR in this specific population is of considerable importance (16). To address this knowledge gap, we conducted a systematic review and meta-analysis to evaluate whether LNR independently predicts survival outcomes in gastric cancer patients who receive neoadjuvant chemotherapy.

## Methods

### Study protocol and methodological adherence

This systematic review and meta-analysis was conducted in strict accordance with the Preferred Reporting Items for Systematic Reviews and Meta-Analyses (PRISMA) guidelines (17). The study protocol was registered with the International Prospective Register of Systematic Reviews (PROSPERO) under the registration number CRD420251233582. All procedures were carried out in accordance with established methodological standards to ensure the rigor of the research process and the transparency of the reporting.

### Information sources and search strategy

We performed a systematic and comprehensive search to identify both published and unpublished literature. Multiple databases were searched from their inception to October 20, 2025, including PubMed, MEDLINE, the Cochrane Central Register of Controlled Trials (CENTRAL), Web of Science, and Embase. To minimize publication bias and identify ongoing studies, our search also encompassed preprint platforms, the ClinicalTrials.gov registry, ahead-of-print publications, and other grey literature (e.g., unpublished research reports and conference abstracts). Furthermore, we conducted backward reference checking of relevant studies and previous systematic reviews, and consulted experts in the field to ensure the breadth and completeness of the search. No restrictions were imposed on language, region, or country to minimize potential selection bias. Three independent reviewers (SL, LS, and XD) performed the literature screening in a blinded manner and resolved discrepancies through discussion. Any unresolved disagreements were adjudicated by a fourth reviewer (KD).Studies were considered eligible if the title, abstract, subject headings, keywords, or unique identifiers included one or more MeSH terms or keywords related to gastric cancer, lymph node ratio, or neoadjuvant treatment. These terms included: “Gastric Neoplasms,” “Stomach Neoplasms,” “gastric cancer,” “lymph node ratio,” “LNR,” “positive lymph node ratio,” “metastatic lymph nodes,” “lymph node metastasis,” “Neoadjuvant Therapy,” “neoadjuvant chemotherapy,” “preoperative chemotherapy,” and “perioperative chemotherapy.”

### Eligibility criteria

This systematic review and meta-analysis was guided by the PICOS framework. The population consisted of patients with histologically confirmed gastric cancer who underwent neoadjuvant chemotherapy—with or without immunotherapy—followed by curative gastrectomy and lymphadenectomy. The exposure of interest was the lymph node ratio (LNR), defined as the ratio of metastatic to examined lymph nodes, assessed either as a categorical or continuous variable. Comparisons included high versus low LNR groups or alternative LNR stratifications based on cut-off values determined in individual studies. The primary outcome was overall survival, while secondary outcome was disease-free survival. Eligible study designs were retrospective cohort studies that reported hazard ratios (HRs) and 95% confidence intervals evaluating the association between LNR and survival outcomes. Randomized clinical trials were not required due to the nature of the research question.

### Study selection

Three reviewers (SL, QH, and XD) independently screened the titles and abstracts retrieved from the systematic search. They then assessed the full texts of studies considered potentially eligible. Any discrepancies during the selection process were resolved through discussion to minimize bias. If consensus could not be reached, a fourth reviewer (KD) made the final determination.

### Data extraction

Three reviewers (SL, QH, and XD) independently extracted data from each included study using a predefined standardized data extraction form. The extracted information included key study characteristics (first author, year of publication, country), patient demographics and clinical details (sample size, age, sex distribution, follow-up duration, and LNR cut-off value), as well as essential information related to neoadjuvant treatment, such as the type of neoadjuvant chemotherapy or immunochemotherapy administered.

In addition, data related to lymph node assessment were collected, including the definitions of lymph node ratio (LNR) used in each study and the corresponding LNR cut-off values. Hazard ratios (HRs) and 95% confidence intervals (CIs) for overall survival, disease-free survival, or other reported survival outcomes were extracted, with priority given to multivariable-adjusted estimates when available. When overall survival was not reported, disease-specific survival was used as an acceptable alternative outcome (17).

A fourth reviewer (KD) independently verified all extracted data to ensure completeness, consistency, and accuracy. Any discrepancies were resolved through discussion until consensus was achieved.

### Risk of bias assessment

The methodological quality of the included cohort studies was independently assessed by three reviewers (SL, QH, and XD) using the Newcastle–Ottawa Scale (NOS) (18). This tool evaluates study quality across three major domains: (1) Selection of study participants, (2) Comparability of cohorts, and (3) Assessment of outcomes. Each study could receive up to a maximum of nine points, with higher scores indicating better methodological quality.

Studies scoring 7–9 points were considered high quality, those scoring 5–6 points were classified as moderate quality, and those scoring below 5 points were deemed low quality. Any discrepancies among reviewers were resolved through discussion, and if consensus could not be reached, a fourth reviewer (KD) adjudicated the final decision. This structured evaluation ensured consistent and rigorous assessment of methodological risk across all included studies.

### Data analysis

Statistical analyses were performed using Review Manager (version 5.4.1) and Stata (version 17.0). For overall survival and disease-free survival, pooled hazard ratios (HRs) with 95% CIs were calculated using the generic inverse-variance method (19). Heterogeneity was evaluated using Cochran’s Q test (P < 0.10 indicating significance) and quantified with I² statistics (20). A random-effects model was applied if significant heterogeneity was observed (P < 0.10 for Q test OR I² ≥ 50%); otherwise, a fixed-effect model was used (21).

### Subgroup analyses

To better understand the potential sources of heterogeneity in the prognostic effect of lymph node ratio after neoadjuvant therapy, subgroup analyses of the primary outcome were conducted based on the following criteria: LNR cutoff (≥0.30 *vs <*0.30) and type of neoadjuvant therapy (chemotherapy *vs* chemo–immunotherapy).The threshold of 0.30 was selected primarily because it was the most frequently employed cutoff across the included studies (10, 22, 23), allowing for the most robust cross-study comparisons, and because it aligns with a value often identified in prior gastric cancer prognosis literature as a critical inflection point for survival outcomes (22).

### Sensitivity analyses

robustness of the pooled OS effect was assessed using leave-one-out sensitivity analyses, repeating the meta-analysis after sequential exclusion of each study (**Table 1**).

**Table 1:** Sensitivity analysis of meta-analysis.

Methods	NO. patients(trials)	HR	95%CI
All trials	2437(7)	3.48	2.27,5.33
Using fixed-effect models	2437(7)	2.97	2.54,3.47
Excluding each randomized control trial in turn			
Excluding Rawicz−Pruszyński et al., 2019 (24)	2342(6)	3.85	2.31,6.39
Excluding Lombardi al. 2021 (22)	2337(6)	4.05	2.55,6.43
Excluding Sakin et al., 2021 (23)	2280(6)	3.79	2.32,6.16
Excluding Zhu et al., 2021 (10)	2172(6)	2.91	2.01,4.21
Excluding Jiang et al., 2022 (25)	2289(6)	3.06	1.99,4.70
Excluding Lai et al.2022 (12)	886(6)	3.81	2.04,7.12
Excluding Zhou et al.2024 (26)	2316(6)	3.32	2.13,5.19

### Assessment of publication bias

Because only seven studies were included, formal assessment of publication bias was not performed. A two-sided p-value < 0.05 was considered statistically significant. However, we acknowledge that the absence of unpublished negative or null-result studies could potentially lead to an overestimation of the pooled effect size. To mitigate this risk, our search strategy explicitly included preprint databases and trial registries.

## Results

### Study selection and characteristics

**Figure 1:**
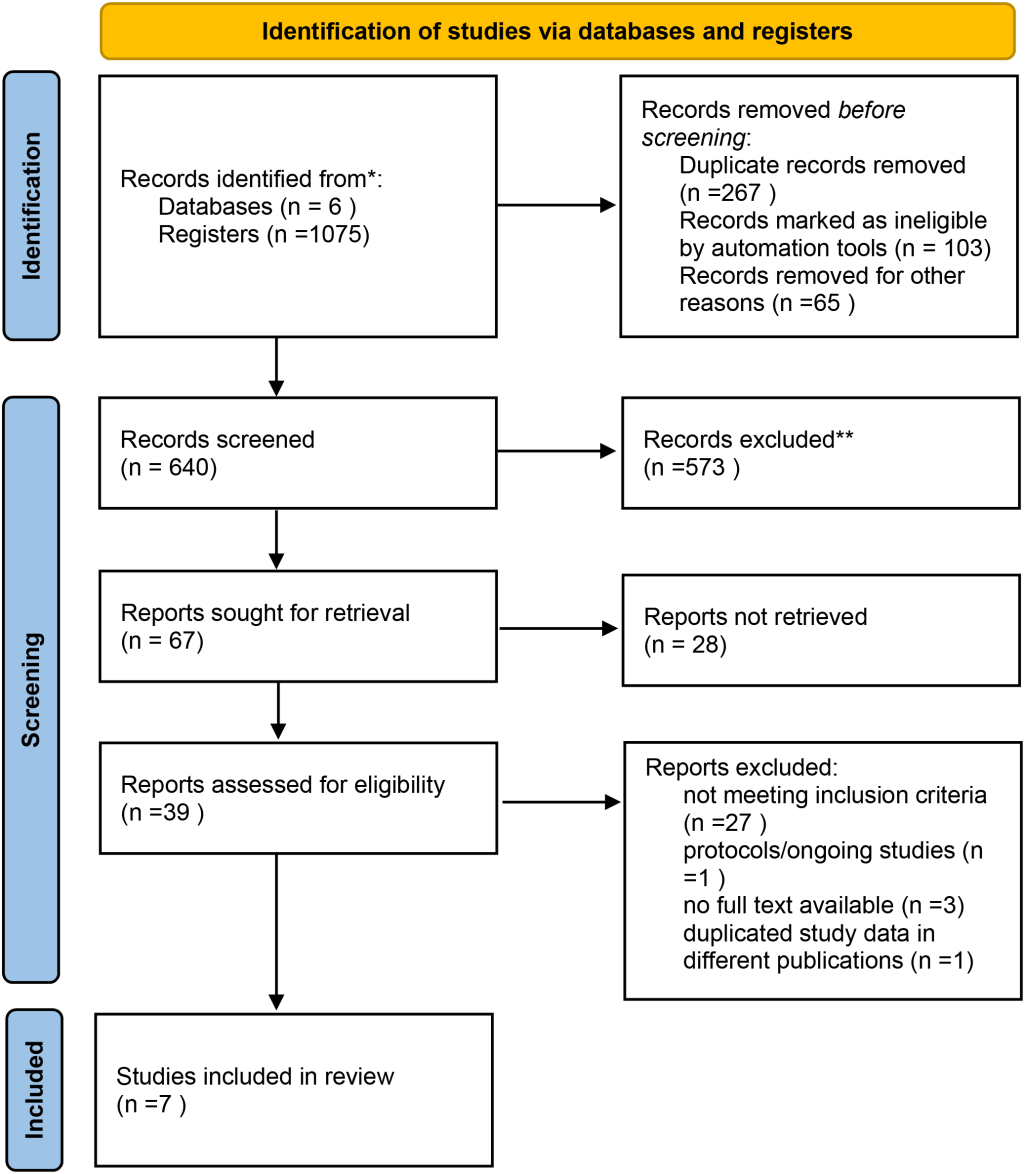
Flow diagram of the included studies.

In the initial search, a total of 1,081 records were identified across all databases. After removing 267 duplicates, 640 unique studies underwent title and abstract screening, of which 573 were excluded. Sixty-seven full-text articles were assessed for eligibility, and 39 were retrieved in full. Following exclusion of studies without neoadjuvant therapy, those lacking lymph node ratio data, unavailable full texts, protocols, or duplicate datasets, seven retrospective cohort studies met the inclusion criteria and were included in the final meta-analysis (**Figure 1**). The seven studies (10, 12, 22–26), published between 2019 and 2024, comprised 2,437 patients with histologically confirmed gastric cancer who underwent neoadjuvant chemotherapy followed by curative gastrectomy. Sample sizes ranged from 95 to 1,551 patients, and the mean or median age ranged from 57 to 65 years. All studies focused on locally advanced gastric adenocarcinoma, with follow-up durations spanning 19 to 42 months. LNR cut-off values varied across studies, including thresholds of 0.10, 0.15, 0.25, 0.26, 0.30, 0.33, 0.40, and 0.50, with 0.30 being the most frequently used. The detailed characteristics of included studies are presented in **Table 2**. Quality assessment using the Newcastle–Ottawa Scale rated seven studies as high quality (scores 7–9), as shown in **Table 3**.

**Table 2:** Main characteristics of the included literature.

Study	Year	Country	Sample size	Research design	Age	Sex(female:male)	NCA	Follow-up	Cutoff LNR	NOS score
Rawicz-Pruszyński	2019	Poland	95	retrospective study	57.37±10.90	Female : Male =41:54	EOX:FLOT=83:12	Median follow-up: 20 months	0.10/0.25/0.50	8
Lombardi	2021	Italy	100	retrospective study	64.81 ± 9.83	Female : Male = 36 : 64	ECF : FLOT = 63 : 37	Median follow-up: 33 months	0.30	8
Sakin	2021	Turkey	157	retrospective study	58(26-75)	female : male = 62 : 95	FLOT :(ECF/ECX/EOF/EOX)= 102 : 55	NA	0.15/0.30/0.45	7
Zhu	2021	China	265	retrospective study	62(34-80)	Female : Male = 72 : 193	FOLFOX : XELOX : SOX = 129 : 43 : 93	42 months	0.1/0.3	9
Jiang	2022	China	148	retrospective study	60(52-64.8)	female:male = 26:122	FOLFOX:SOX:XELOX:FLOT = 81:55:7:5	34.6 months	0.26	9
Lai	2022	US SEER	1551	retrospective study	≤65: 962;>65: 589	Female : Male = 395 : 1156	unclear	27 months	0.40	9
Zhou	2024	China	121	retrospective study	58.3 ± 9.4	Female : Male = 26 : 95	Tislelizumab:Nivolumab:Sintilimab:Toripalimab =80:7:15:19	19 months	0.33	8

**Table 3:** Quality of the included studies.

Study	Representativeness	Selection of non-exposed	Ascertainment of exposure	Outcome not present at start	Comparability (main factors)	Comparability (other factors)	Assessment of outcome	Long enough follow-up	Adequacy of follow-up	Total
Rawicz-Pruszyński 2019 (24)	✓	✓	✓	✓	✓	✓	✓	✓	✓	8
Lombardi 2021 (22)	✓	✓	✓	✓	✓	✓	✓	✓	✓	8
Sakin 2021 (23)	✓	✓	✓	✓	✓	×	✓	×	✓	7
Zhu 2021 (10)	✓	✓	✓	✓	✓	✓	✓	✓	✓	9
Jiang 2022 (25)	✓	✓	✓	✓	✓	✓	✓	✓	✓	9
Lai 2022 (12)	✓	✓	✓	✓	✓	✓	✓	✓	✓	9
Zhou 2024 (26)	✓	✓	✓	✓	✓	✓	✓	×	✓	8

### Primary outcome and secondary outcome

**Figure 2:**
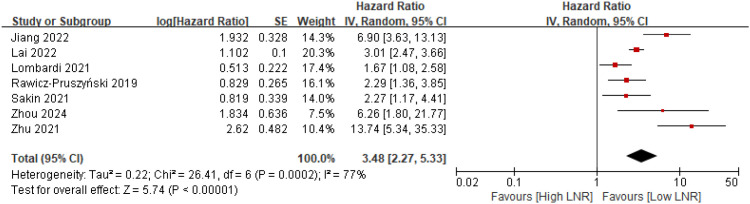
Forest plot comparing overall survival between high and low lymph node ratio groups after neoadjuvant therapy for gastric cancer.

**Figure 3:**
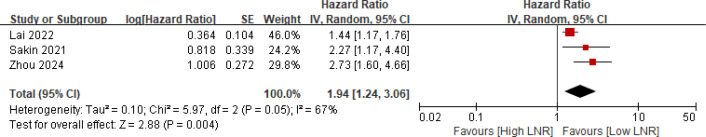
Forest plot comparing disease-free survival between high and low lymph node ratio groups after neoadjuvant therapy for gastric cancer.

Six studies (10, 12, 23–26) reported overall survival (OS), while one study (22) provided disease-specific survival (DSS) as an alternative outcome. The pooled analysis demonstrated that patients with a high lymph node ratio experienced significantly poorer overall survival compared with those with a low lymph node ratio, with a combined hazard ratio of 3.48 (95% CI 2.27–5.33) (**Figure 2**). Three studies (12, 23, 26) reported disease-free survival, and high LNR was consistently associated with inferior DFS, yielding a pooled hazard ratio of 1.94 (95% CI 1.24–3.06) (**Figure 3**).

### Subgroup analyses

**Figure 4:**
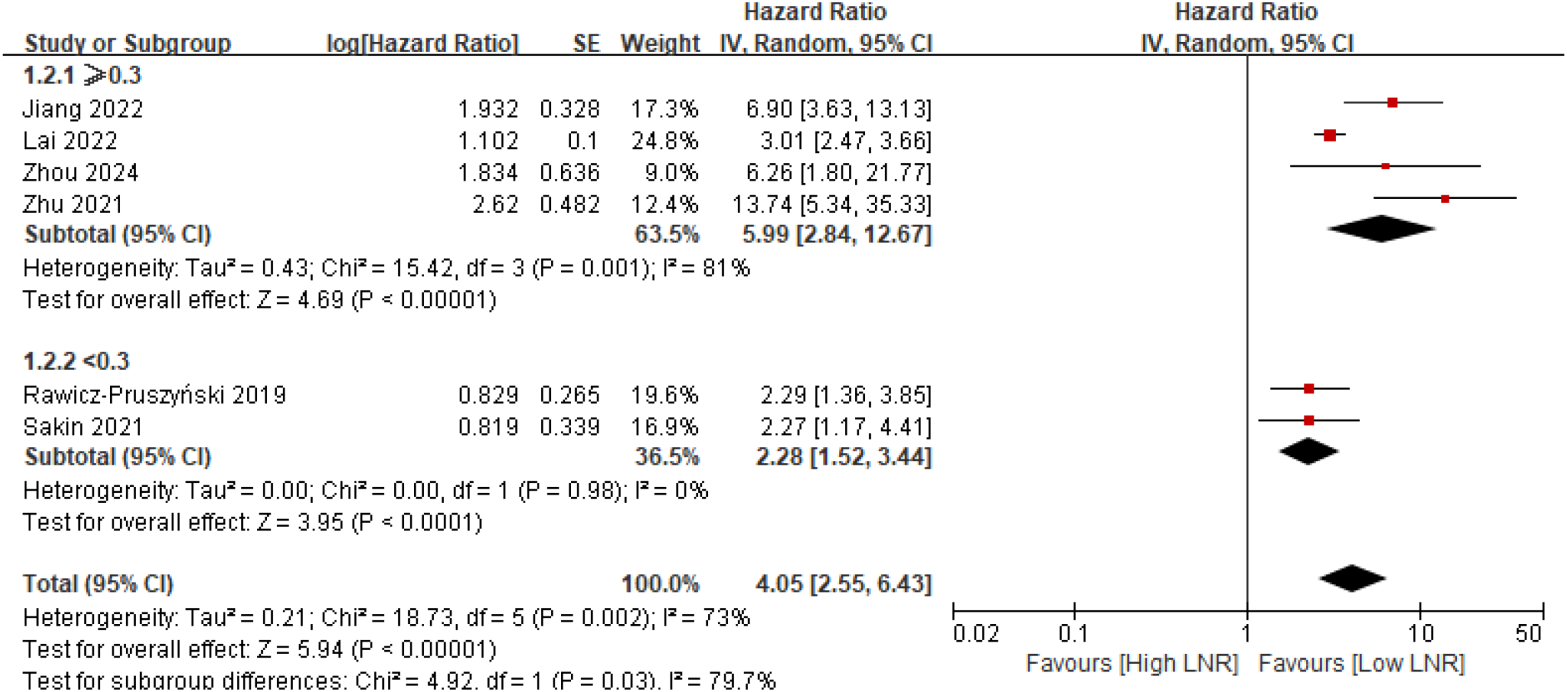
Subgroup analysis of overall survival stratified by lymph node ratio cut-off of 0.30 in gastric cancer patients treated with neoadjuvant therapy.

**Figure 5:**
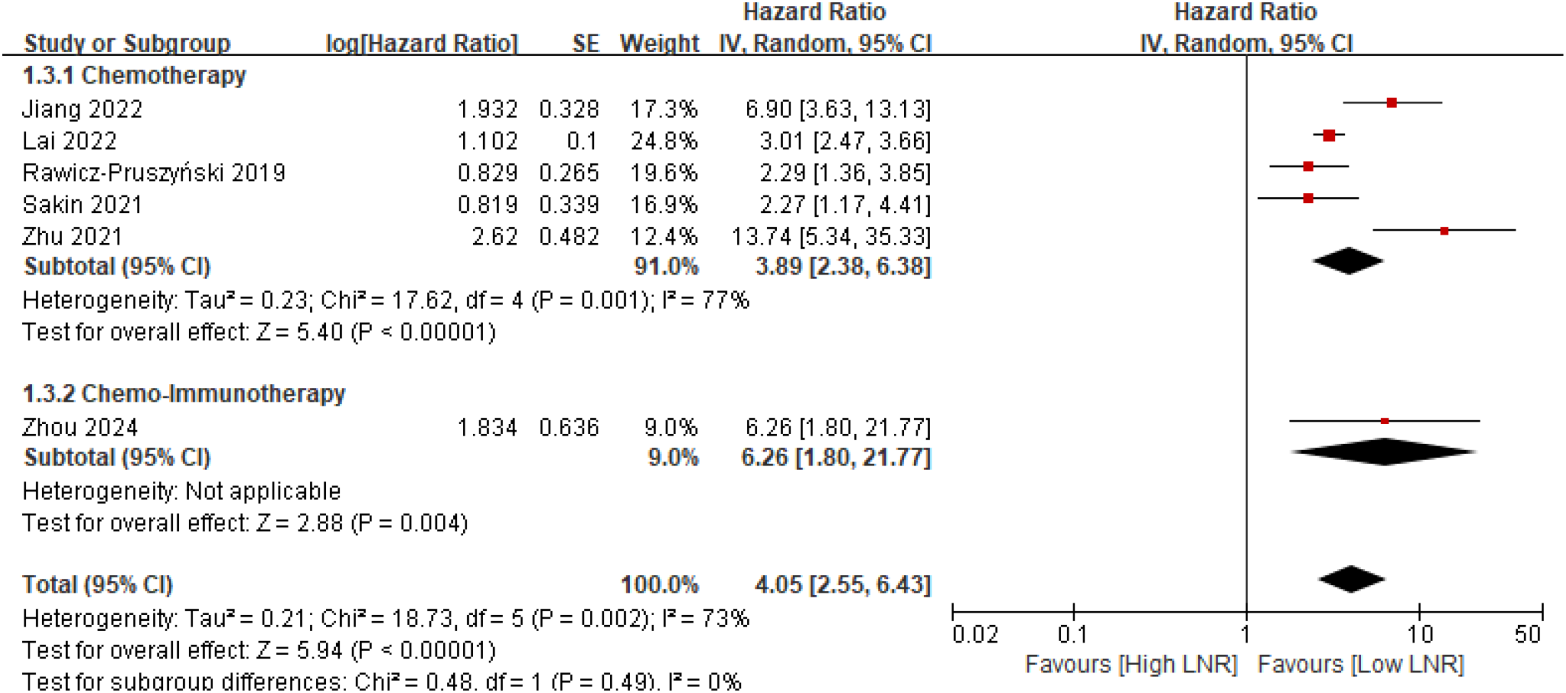
Subgroup analysis of overall survival stratified by type of neoadjuvant therapy (chemotherapy versus chemo-immunotherapy) in gastric cancer patients.

Subgroup analysis based on the commonly used LNR threshold of 0.30 revealed a statistically significant interaction between studies using cut-offs ≥0.30 and those using cut-offs <0.30 (p for interaction = 0.03) (**Figure 4**). In contrast, subgroup analysis stratified by neoadjuvant treatment modality demonstrated no statistically significant interaction between chemotherapy-only regimens and chemo–immunotherapy regimens (p for interaction = 0.49) (**Figure 5**).

### Sensitivity analyses

Sensitivity analyses confirmed the robustness of these findings, with leave-one-out pooled hazard ratios ranging from 2.91 to 4.05, all remaining statistically significant, and with consistent results across fixed- and random-effects models (**Table 1**).

## Discussion

This systematic review and meta-analysis included seven retrospective cohort studies (10, 12, 23–26) comprising 2,437 patients with locally advanced gastric cancer who underwent neoadjuvant chemotherapy followed by curative gastrectomy. Across all included studies, we observed that a higher lymph node ratio (LNR) was consistently associated with a markedly increased risk of death and inferior survival outcomes.

Our study indicates that an elevated lymph node ratio (LNR) is significantly associated with multiple survival outcomes in patients with gastric cancer. This finding is consistent with previous meta-analyses (14, 15) and corroborates earlier research demonstrating that LNR serves as an independent prognostic marker across various cancer. However, earlier meta-analyses (27) primarily included patients undergoing upfront surgery and explicitly excluded those who received neoadjuvant chemotherapy. Given that neoadjuvant treatment may alter lymph node architecture through fibrosis (28), sterilization of metastatic deposits, or reductions in lymph node yield (10), the prognostic performance of LNR in this setting cannot be assumed. Therefore, it is essential to reassess the prognostic value of LNR in the context of neoadjuvant therapy, and the present study provides evidence addressing this important clinical question.

Although the mechanisms underlying why LNR remains a strong prognostic indicator after neoadjuvant therapy are not yet fully understood, several plausible explanations may account for this observation. First, LNR reflects both the true residual metastatic burden and the adequacy of lymph node evaluation, thereby mitigating the stage-migration effect commonly observed in pathological nodal status after neoadjuvant therapy (12). Second, neoadjuvant chemotherapy induces pathological changes like intranodal fibrosis and lymph node atrophy (29). Fibrosis can obscure metastatic deposits, complicating histologic detection, while treatment-related shrinkage often reduces total lymph node yield (30). These alterations undermine conventional nodal staging, which depends on absolute positive node counts (31). In contrast, the lymph node ratio, as a proportional measure, inherently adjusts for variations in node retrieval (32). By integrating residual metastatic burden with the extent of nodal evaluation, LNR mitigates the confounding effects of therapy-induced changes and offers a more stable representation of true disease burden (32, 33). Third, residual lymph node metastasis following systemic therapy often indicates more aggressive tumor biology and an incomplete treatment response, and LNR is particularly sensitive to capturing this residual disease burden (34). Fourth, LNR integrates multiple dimensions, including tumor load, treatment response, and surgical quality. Therefore, it provides a more comprehensive and stable prognostic assessment than staging systems based solely on the absolute number of positive lymph nodes (12). Taken together, these factors may explain why LNR continues to demonstrate significant and clinically meaningful prognostic value in the post-neoadjuvant setting.

This study has several limitations. First, All included studies were retrospective, with inherent risks of selection bias and residual confounding. Additionally, the relatively small number of included studies (n=7) and total patients (n=2,437) may limit the generalizability of our findings and the statistical power for certain subgroup analyses. Second, there was considerable variability in the lymph node ratio (LNR) cutoff values applied across the studies, ranging from 0.10 to 0.50. This heterogeneity may affect the generalizability of the pooled hazard ratios and complicates the establishment of a standardized risk threshold. Our subgroup analysis indicated a significant interaction at the 0.30 cutoff (p=0.03), suggesting that higher thresholds may provide more stable prognostic value in the post-neoadjuvant chemotherapy setting; however, prospective studies are required to determine the optimal LNR cutoff. Furthermore, for some studies, survival data had to be extracted from published survival curves, a process which may introduce minor measurement inaccuracies. Finally, the relatively small number of studies reporting disease-free survival (DFS) limited our capacity to robustly assess other secondary endpoints.

The clinical implications of our findings are notable. High LNR identifies a subgroup of patients at markedly elevated risk of recurrence and death after neoadjuvant therapy, indicating that these individuals may benefit from intensified postoperative management, closer surveillance, or alternative adjuvant strategies. Furthermore, LNR may complement the current pathological Tumor–Node–Metastasis staging system staging after neoadjuvant therapy improving risk stratification accuracy in the neoadjuvant era and supporting more individualized postoperative decision-making. Despite these limitations, this meta-analysis represents the most comprehensive synthesis to date examining the prognostic value of LNR after neoadjuvant chemotherapy in gastric cancer. The consistent findings across multiple cohorts and analytical approaches underscore the strong prognostic significance of LNR in modern clinical practice and highlight the need for future prospective studies to determine how LNR can be integrated into postoperative staging systems and adjuvant treatment decision pathways (2–6). Specifically, future research should prioritize the design of multicenter, prospective validation cohort studies. These studies ought to employ standardized pathological assessment protocols for lymph nodes—including recommended minimum yield thresholds and harmonized lymph node retrieval and dissection methods—to minimize heterogeneity and validate optimal LNR risk stratification cut-offs. Furthermore, exploring the integration of LNR with other pathological or molecular biomarkers holds significant value for developing more accurate multivariate prognostic prediction models.

## Conclusion

Higher LNR is a strong and independent predictor of poor survival after neoadjuvant chemotherapy in gastric cancer. LNR may improve postoperative risk stratification beyond pathological Tumor–Node–Metastasis staging system staging after neoadjuvant therapy, but prospective studies are required to establish optimal cut-offs and determine how LNR should be integrated into clinical decision-making.

## Data Availability

The raw data supporting the conclusions of this article will be made available by the authors, without undue reservation.
